# Suicide gene therapy for hepatocellular carcinoma cells by survivin promoter-driven expression of the herpes simplex virus thymidine kinase gene

**DOI:** 10.3892/or.2013.2248

**Published:** 2013-01-24

**Authors:** LILI QU, YANYUN WANG, LAILING GONG, JIN ZHU, RUJUN GONG, JIN SI

**Affiliations:** 1 Department of Laboratory Medicine, The Second Affiliated Hospital of Nanjing Medical University, Nanjing 210011, P.R. China; 2 Huadong Medical Institute of Biotechniques, Nanjing 210002, P.R. China; 3 Department of Medicine, Brown Medical School, Providence, RI 02903, USA

**Keywords:** hepatocellular carcinoma, survivin, gene therapy, thymidine kinase/ganciclovir

## Abstract

The aim of this study was to investigate the selective killing effect of the herpes simplex virus-thymidine kinase/ganciclovir (TK/GCV) suicide gene system controlled by the survivin promoter on hepatocellular carcinoma (HCC) cells *in vitro*. Recombinant plasmid vectors driven by the survivin promoter were constructed. HepG2 HCC and LO2 normal human liver cells were transfected with the recombinant plasmids, green fluorescent protein (GFP)/pSURV, TK/pSURV and TAT-TK/pSURV. GFP expression was detected by fluoroscopy and flow cytometry (FCM). TK gene expression was detected using RT-PCR and western blot analysis. The selective killing effects after GCV application were evaluated by tetrazolium assay, FCM and western blot analysis. Statistical analysis was performed by ANOVA. After transfection with GFP/pSURV, TK/pSURV and TAT-TK/pSURV for 48 h, GFP expression was observed in the HepG2 cells, but not in the L02 cells and TK gene expression was evidently detected by RT-PCR and western blot analysis in the HepG2 cells. Three stably transfected cell lines (HepG2/pSURV, HepG2/TK/pSURV and HepG2/TAT-TK/pSURV) were successfully established. Compared with the HepG2/TK/pSURV group, a significant ‘bystander effect’ was observed in the HepG2/TAT-TK/pSURV group with the incorporation of unmodifed HepG2 cells at different ratios. Following transfection with TK/pSURV and TAT-TK/pSURV, the growth of HepG2 cells in the presence of GCV was markedly inhibited. This finding was further corroborated by FCM and immunoblot analysis revealed the repressed expression of proliferating cell nuclear antigen (PCNA). Our results showed that the plasmid vectors carrying the TK and TAT-TK fusion protein gene driven by the survivin promoter were successfully constructed and their specific expression in HepG2 cells provided the basis for the targeted gene therapy of HCC.

## Introduction

Hepatocellular carcinoma (HCC) is the fifth most common malignant tumor worldwide, and the third most common cause of cancer-related mortality (1–4). Chemotherapy, as a treatment for HCC patients has proven to be toxic and not very effective. The most effective therapy to date is still surgical resection; however, less than 15% of patients benefit from this treatment due to the presence of multiple tumor nodules. This situation is slowly being improved with a better understanding of the molecular mechanisms involved in the pathogenesis of HCC. It is imperative to develop novel strategies, such as suicide gene therapy, in which nucleic acids encoding specific therapeutic genes are used as anti-tumor agents.

The best characterized suicide gene therapy system is the herpes simplex virus type 1 thymidine kinase (TK) combined with the guanosine analog, ganciclovir (GCV), which is phosphorylated with the aid of TK into a GCV monophosphate and further by cellular kinases into di- and triphosphate forms (5). GCV triphosphate is incorporated into DNA during cell division, causing single-strand DNA breaks and the inhibition of DNA polymerase (6–8), thus causing DNA chain termination, eventually leading to programmed cell death. Moreover, nearby unmodified tumor cells are known to also be killed by the ‘bystander effect’ in this form of therapy. Both the transfection efficiency and ‘bystander effect’ are essential factors for the success of the anti-tumor efficacy of HSV-TK and prodrug GCV suicide gene therapy system. The potential of the HIV-1 transactivator protein transduction domain (TAT PTD) as a carrier to deliver various macromolecules into cells has been established in a number of recent studies. In the majority of the studies, the TAT peptide has been utilized to cargo different proteins and peptides mostly in cultured cells but also in animal models (9). In the field of gene therapy, the TAT peptide has been harnessed to, for example, enhance viral vector transduction efficiency (10,11) and gene-transfer efficiency of liposomes (12).

However, the clinical efficacy of gene therapy is not satisfactory due to the lack of targeting (13). Survivin, a member of the inhibitor of apoptosis (IAP) family, has attracted considerable attention as an ideal target for cancer treatment since it is highly and uniquely expressed in the majority of human tumors and plays a critical role in the control of cell division and the inhibition of apoptosis (14–18). A high level of survivin expression has been observed in HCC (19). In the present study, we constructed the TK and TAT-TK gene expression vector with the promoter of the survivin gene and examined its effect on the inhibition of proliferation and the induction of apoptosis in the HCC cell line, HepG2.

## Materials and methods

### Cell culture and DNA preparation

The L02 human liver cell line and the HepG2 human HCC cell line were purchased from KeyGen. Cells were cultured in DMEM (Gibco, Carlsbad, CA, USA) supplemented with 10% newborn calf serum (Gibco) at 37°C in a humidified atmosphere containing 5% CO_2_.

### Construction of expression vector

**Figure 1:**
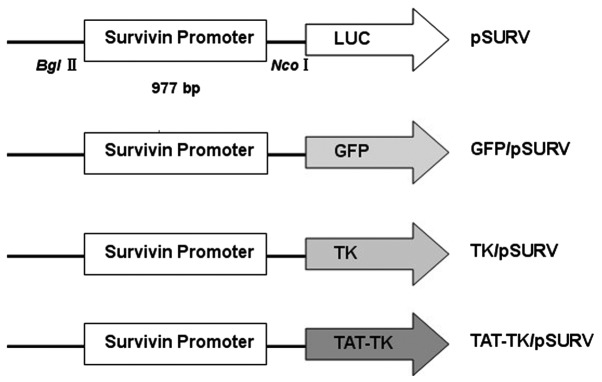
Plasmid constructs. In the luciferase reporter plasmids, the promoters were cloned upstream of the luciferase gene in the restriction sites. GFP/pSURV, TK/pSURV and TAT-TK/pSURV were constructed by replacing the luciferase gene with the GFP, TK and TAT-TK gene. Luc, luciferase gene.

Four different plasmids were constructed (Fig. 1). In order to create the luciferase expression plasmid under the control of the survivin promoter (pSURV-Luc), we generated a 977-bp fragment of the human survivin gene promoter (nucleotides 1824–2800, GenBank Accession no. U75285) by polymerase chain reaction (PCR) of human bacterial artificial chromosome libraries (RPC 1–11, 0219G17; ResGen, Inc., San Leandro, CA, USA) as a template. The sequences of the oligonucleotides primers were: forward primer, 5′-ATACGAGATCTGCCATAGAACCA-3′ and reverse primer, 5′-ATGTAAAGCTTCCACCTCTGCCA-3′ (20). After restriction enzyme digestion and purification, the fragment was inserted into the luciferase vector, pGL3-basic (Promega Corp., Madison, WI, USA), at the *Bgl*II and *Hin*dIII sites. To create the green fluorescent protein (GFP), TK and TAT-TK constructs, termed GFP/pSURV, TK/pSURV and TAT-TK/pSURV, we replaced the luciferase gene of pSURV-Luc with GFP, TK and TAT-TK, respectively by *Nco*I and *Xba*I digestion. The GFP fragment was previously preserved in our laboratory. The TK fragment from the TK gene-containing plasmid, r-pAs16Dr (Professor A. Söling, Martin-Luther-University Halle-Wittenberg, Halle, Germany), was amplified using the following primers: forward, 5′-ATCCATGGATGGCTTCGT ACCCCT-3′; and reverse, 5′-ATCTCGAGTCAAGCCTCCC CC-3′. The TAT-TK fragment from the TAT-TK gene-containing plasmid, pGEX2T-Tat11-TK [Professor M. Zoppè, International Center for Genetic Engineering and Biotechnology (ICGEB), Trieste, Italy], was amplified using the forward primer, 5′-ATC CATGGGCATGTATGGCAGGAAG-3′; and the reverse primer, 5′-GCTCTAGACGGAGGACAGTCCTCCGGAGA CCGGAGGACAGTCCTCCGTCAGTTAGCCTCC-3′. These plasmids were confirmed by sequence analysis.

### Transient transfection and GFP reporter assays

Cells were plated in six-well plates at a density of 3×10^5^ cells per well and incubated overnight. Cells were transfected with GFP/pSURV using X-tremeGENE HP DNA Transfection Reagent (Roche, USA) according to the manufacturer’s instructions. After 48 h of transfection, the GFP/pSURV tansfection efficiency of HepG2 and L02 cells were detected by fluorescence microscopy and flow cytometry (FCM).

### Stable transfection

After transfection for 48 h, HepG2 cells were diluted to 1:10 for passage and neomycin-resistant clones were selected in the medium containing 400 μg/ml G418 (Gibco) for two weeks. The positive clones were selected and expanded to establish cell lines. The stably transfected cell clones, designated as HepG2/pSURV, HepG2/TK/pSURV and HepG2/TAT-TK/pSURV, were verified by quantitative real-time RT-PCR and western blot analysis.

### Quantitative real-time reverse transcriptase (RT)-PCR

Total cellular RNA was extracted using TRIzol reagent (Invitrogen, Carlsbad, CA, USA). RNA integrity was confirmed by electrophoresis on ethidium bromide-stained 1% agarose gels. The primer sequences used for TK were: sense, 5′-TGGCCAAA CGCCTCCGTTCC-3′ and antisense, 5′-GTGCGCGCCA GGTCACATA-3′; GAPDH: sense, 5′-TAAATTGAGCCCG CAGCCTCCC-3′ and antisense, 5′-GACCAAATCCGTT GACTCCGACCT-3′. The mRNA level for TK was analyzed by one-step real-time RT-PRC with RNA-direct™ SYBR-Green Real-time PCR Master mix (Toyobo, Osaka, Japan) according to the manufacturer’s instructions. Cycling conditions were: 90°C for 30 sec, 61°C for 20 min, 95°C for 60 sec, then 40 cycles at 95°C for 15 sec and 60°C for 1 min. The amplification was monitored on an ABI Prism 7500 Real-time PCR apparatus (Applied Biosystems, USA). The products were detected on a 1% agarose gel.

### Western blot analysis

Cells were harvested from flasks, and lysed with ice-cold lysis buffer (50 mM Tris-HCl, pH 7.4, 150 mM NaCl, 1 mM MgCl_2_, 100 μg/ml PMSF and 1% Triton X-100 ) for 30 min on ice. Cell lysates were then collected after centrifugation at 12,000 rpm for 5 min at 4°C. Equal amounts (40 μg) of lysate proteins were separated on 10% SDS-PAGE gels, and transblotted onto PVDF membranes (Pall Corp., Ann Arbor, MI, USA). After blocking with 5% non-fat dry milk in TBST buffer (10 mM Tris, pH 7.5, 150 mM NaCl, and 0.05% Tween-20) for 2 h at room temperature, the membranes were probed with 1:500 dilution of anti-TK (Santa Cruz Biotechnology, Inc., Santa Cruz, CA, USA) or anti-β-actin (Sigma, St. Louis, MO, USA) antibodies at 4°C overnight, followed by incubation in a 1:1,000 dilution of secondary antibodies conjugated to horseradish peroxidase (the membranes were probed with a 1:500 dilution of anti-TK (Santa Cruz Biotechnology, Inc.) or anti-β-actin (Sigma) for 1 h at room temperature. Protein bands were detected using the ECL detection system. All the western blot analyses were performed at least three times.

### Cell proliferation assay

Cell proliferation was analyzed with 3-(4, 5-dimethylthiazol-2-yl)-2, 5-diphenyltetrazolium bromide (MTT; Sigma). Briefly, 8,000 cells from each group were plated per well in three 96-well microplates in 200 μl of medium and replaced with 0, 5, 10, 20, 40, 60, 80 and 100 μg/ml GCV the following day. After 48 h, 50 μl of MTT substrate were added to each well, and the plates were returned to standard tissue incubator conditions for an additional 4 h. The medium was then removed, the cells were solubilized in 150 μl of dimethyl sulfoxide, and colorimetric analysis was performed (wavelength, 490 nm). The inhibition rate was calculated as [1-(OD value of the transfection/OD value of untreated HepG2 cells)] ×100%. Each experiment was performed in triplicate.

### ‘Bystander effect’ analysis

The modified cells, HepG2/TK/pSURV and HepG2/TAT-TK/pSURV, incorporated with HepG2 unmodified cells at different ratios (0, 10, 20, 40, 80 and 100%), adjusted to a density of 8,000 cells/well, were co-cultured in 96-well plates at 37°C for 48 h in the presence of 20 μg/ml of GCV. The ‘bystander effect’ was determined using the MTT method, as described above.

### Apoptosis detection by FCM

The apoptotic cells were differentiated from viable or necrotic ones by combined application of Annexin V-FITC and propidium iodide (PI) (BD Biosciences, Franklin Lakes, NJ, USA). The samples were washed twice and adjusted to a concentration of 5×10^5^ cells/ml with PBS. Suspensions (200 μl) was added to each labeled tube, 5 μl of Annexin V-FITC and 10 μl PI (20 μg/ml) were added into the labeled tube, incubated for at least 10 min at room temperature in the dark, then 200 μl of PBS binding buffer were added to each tube without washing and analyzed using FCM (BD Biosciences) as soon as possible (within 30 min). Apoptotic cells were defined as the population that were PI-negative (indicating an intact plasma membrane) and Annexin V-FITC-positive. This assay was performed in triplicate.

### DNA fragmentation analysis

Cells (1×10^4^ cells) were centrifuged, resuspended in 200 μl PBS, incubated with 4 μl RNase A for 0.5 h at 37°C, followed by digestion with 20 μl proteinase K for 1 h at 37°C, and then lysed in 200 μl of lysis buffer [1 ml of 1 M Tris-HCl buffer at pH 7.4, 0.2 ml of 0.5 M ethylenediaminetetraacetic acid (EDTA)], and 0.5 ml 10% Triton X-100). Lysed cells were held at 70°C for 10 min. The solution was mixed with 5 M NaCl (20 μl) and isopropanol (120 μl), and the mixture was incubated at −20°C for 24 h, followed by centrifugation at 15,000 × g for 20 min. The precipitated DNA was dissolved in Tris-EDTA (5 μl) buffer and subjected to electrophoresis using a 1% agarose gel and Tris-acetate-EDTA buffer at 90 V. The DNA fragmentation pattern was visualized with a UV transilluminator.

### Detection of apoptosis and proliferation signaling by western blot analysis

Western blot analysis were performed as described above. Rabbit polyclonal antibodies against cleaved caspase-3 (Cell Signaling, USA) and proliferating cell nuclear antigen (PCNA) (Santa Cruz Biotechnology, Inc.) were employed for antigen detection while rabbit polyclonal antibody against GAPDH (Xianzhi Bio, Hangzhou, China) was used as the control. Immunodetection was carried out with HRP-coupled secondary antibodies to mouse (Sigma) or rabbit (Santa Cruz Biotechnology, Inc.) antibodies.

### Statistical analysis

SPSS16.0 software was used. Each assay was performed at least three times. The data are expressed as the means ± SD. The Student’s t-test and ANOVA were used to determine the significance of differences in multiple comparisons. P<0.05 was considered to indicate a statistically significant difference.

## Results

### Cancer-specific transgene expression of the survivin promoter in vitro

**Figure 2:**
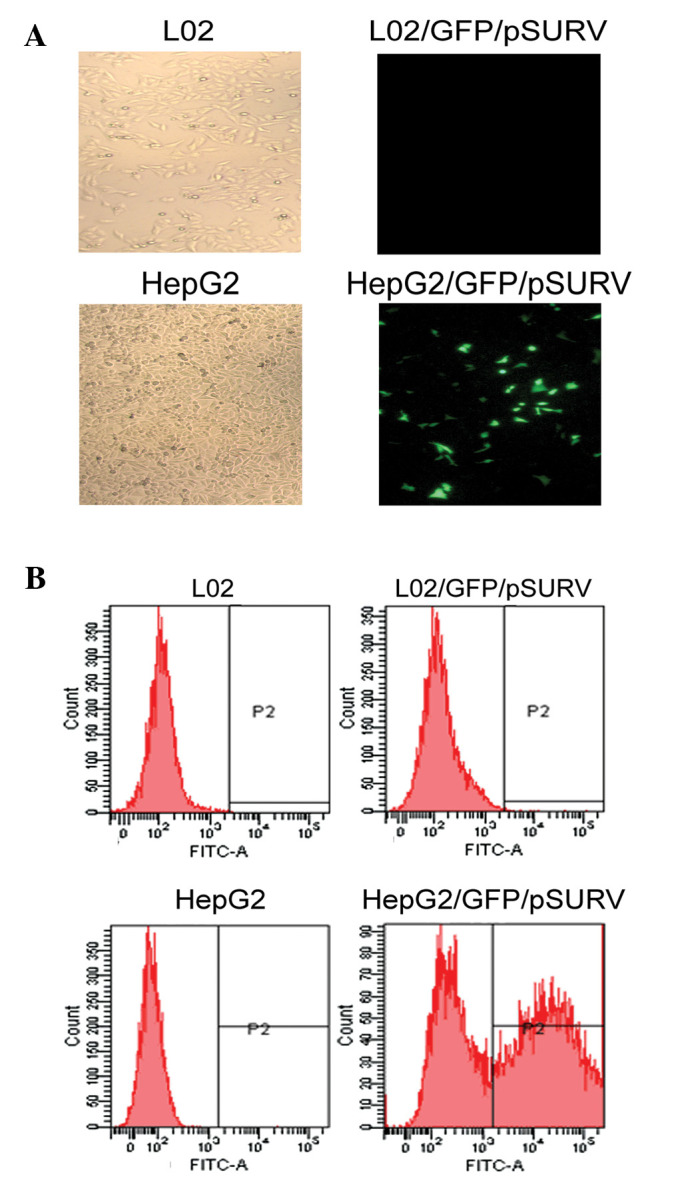
(A) LO2 and HepG2 cell lines transfected with GFP/pSURV observed under a fluorescence microscope (x50). (B) LO2 and HepG2 cell lines transfected with GFP/pSURV. Transfection efficienty was detected by flow cytometry. The activity of the survivin promoter was much higher in the HepG2 (50.3%) compared to the LO2 cells (0.3%).

To assess whether the activation of the survivin promoter is cancer-specific, we used GFP/pSURV to determine the activity of the survivin promoter in the HepG2 cancer cell line and in the L02 normal hepatic cell line. As shown in Fig. 2, a stronger fluorescence signal was observed in the HepG2 cells, 48 h after transfection with GFP/pSURV, indicating that the survivin promoter was more specifically activated in the HCC cells than in the normal cells.

### Survivin promoter-driven expression of TK

**Figure 3:**
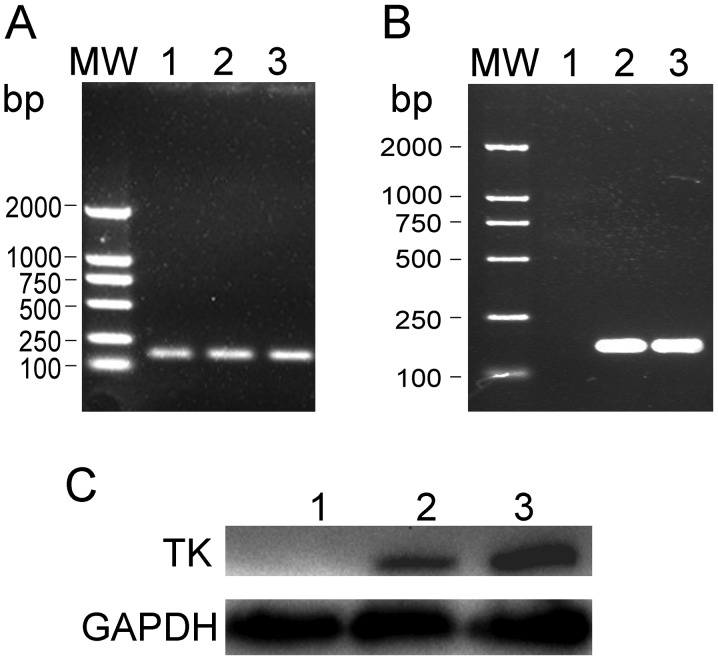
Expression of TK after HepG2 cell transfection. (A) GAPDH bands of 140 bp were detected in the HepG2, HepG2/TK/pSURV and HepG2/TAT-TK/pSURV groups and (B) TK bands of 158 bp were only detected in the HepG2/TK/pSURV and HepG2/TAT-TK/pSURV groups. (C) The TK protein was detected in the HepG2/TK/pSURV and HepG2/TAT-TK/pSURV groups. Lane 1, HepG2; lane 2, HepG2/TK/pSURV; lane 3, HepG2/TAT-TK/pSURV.

To determine whether the recombinant plasmids, TK/pSURV and TAT-TK/pSURV, can be successfully transferred into HepG2 cells and whether the TK expression can be achieved, total HepG2 cellular RNA and protein were extracted from the different transfected groups. The expression of TK was detected by RT-PCR and western blot analysis. As shown in Fig. 3, TK was strongly expressed in the HepG2/TK/pSURV and HepG2/TAT-TK/pSURV groups, but not in the HepG2 and HepG2/pSURV groups.

### Expression of TK driven by the survivin promoter inhibits cancer cell growth in vitro

**Figure 4:**
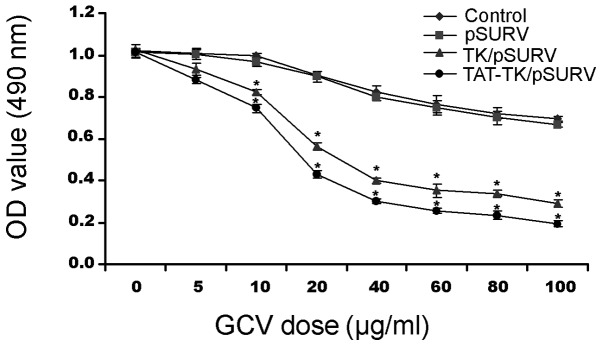
GCV dose-response curves for HepG2 (control), HepG2/pSURV, HepG2/TK/pSURV and HepG2/TAT-TK/pSURV. A significantly higher growth inhibition was observed in the HepG2/TK/pSURV and HepG2/TAT-TK/pSURV groups compared to the other groups. (P<0.05).

To investigate the biological effect induced by these novel suicide gene systems (TK/pSURV and TAT-TK/pSURV), the level of cell proliferation was assessed by MTT assay. As shown by the cell growth inhibition rate curve (Fig. 4), the cell growth inhibition rates in the HepG2/TK/pSURV and HepG2/TAT-TK/pSURV groups gradually increased and the cell growth inhibition rate in the HepG2/TAT-TK/pSURV group was higher than that in the HepG2/TK/pSURV group under the same experimental conditions (P<0.05). No difference in cell proliferation was observed between the HepG2 and HepG2/pSURV groups.

### ‘Bystander effect’ in the presence of TK/pSURV/GCV and TAT-TK/pSURV/GCV

**Figure 5:**
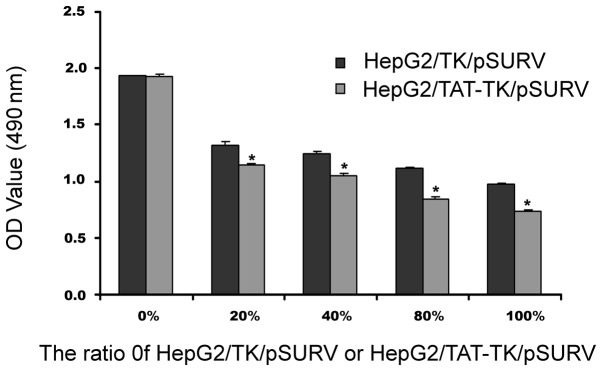
‘Bystander effect’ assay.

As shown in Fig. 5, with the increase in the percentage of TK/pSURV/GCV- or TAT-TK/pSURV/GCV-transfected cells co-cultured with unmodifed HepG2 cells, cell growth inhibition rates were progressively elevated, indicative of a marked ‘bystander effect’. However, the most prominent ‘bystander effect’ was observed in the HepG2/TAT-TK/pSURV group.

### Suicide gene system induces apoptosis in HepG2 cells

**Figure 6:**
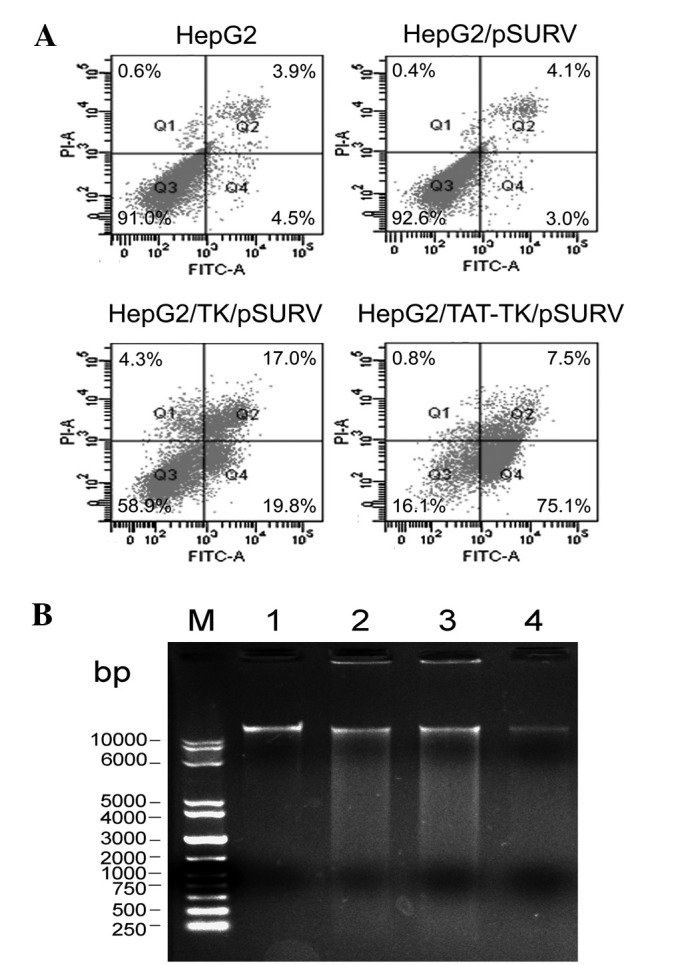
Analysis of HepG2 cell apoptosis. (A) Flow cytometric detection of HepG2 cell apoptosis. The points of the lower right quadrant represent apoptotic cells, and the points of the upper right quadrant represent necrotic cells. (B) Formation of DNA ladder. (M, molecular weight; lane 1, HepG2; lane 2, HepG2/TK/pSURV; lane 3, HepG2/TAT-TK/pSURV; lane 4, HepG2/pSURV).

Based on Annexin V-FITC and PI staining, FCM was applied to quantify the extent of apoptosis. As demonstrated in Fig. 6A, the apoptotic rates in the HepG2/TK/pSURV and HepG2/TAT-TK/pSURV groups were 20.51 and 55.82%, respectively, which were significantly higher compared to the other groups (P<0.05). The maximal apoptotic rate in the HepG2/TAT-TK/pSURV group was significantly higher than that in the HepG2/TK/pSURV group (P<0.05). The findings observed by FCM were corroborated by DNA fragmentation analysis (Fig. 6B).

### Suicide gene system modifies cell proliferation and apoptosis signaling activity

**Figure 7:**
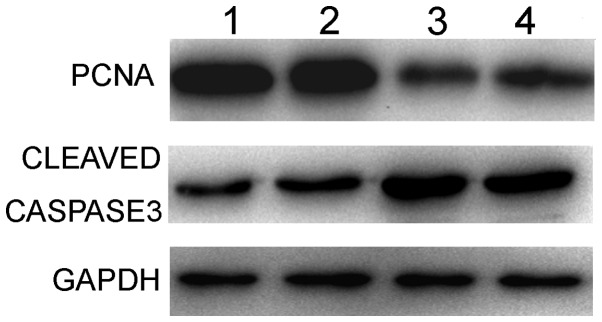
Detection of cleaved caspase-3 and PCNA activation. Lane 1, HepG2; lane 2, HepG2/pSURV; lane 3, HepG2/TK/pSURV; lane 4, HepG2/TAT-TK/pSURV.

The PCNA protein levels and active caspase-3 were subsequently detected. As shown in Fig. 7, the expression of PCNA in the HepG2/TK/pSURV and HepG2/TAT-TK/pSURV groups was lower compared to the HepG2 and HepG2/pSURV groups, while that of caspase-3 was higher compared to the other groups. These findings strongly suggest that the TAT-TK/GCV gene therapy system mechanistically upregulates caspase-3 directed apoptotic signaling and represses PCNA-mediated cell proliferation in HepG2 cells.

## Discussion

Despite other recent advances in anticancer therapeutics, tumor-specific suicide gene therapy using a tissue-specific promoter is a rational therapeutic strategy for HCC. Suicide gene therapy using the herpes simplex virus thymidine kinase/ganciclovir (HSV-TK/GCV) system is a well-characterized tool used in cancer gene therapy (21–25). However, thus far, this suicide gene system (HSV-TK/GCV) has demonstrated little efficacy in clinical practice, mostly due to low targeting, absence of the ‘bystander effect’ and poor gene-transfer efficiency in tumors.

Survivin, a member of the IAP family is overexpressed in most types of cancer cells and embryonic tissue; however, it is only slightly detected in terminally differentiated normal tissue (26). Transcription experiments have indicated that the protein expression of survivin in cancer tissue appears to be regulated, at least in part, transcriptionally (27,28). Since the increased survivin activity is controlled transcriptionally, it has been suggested that the survivin promoter may control the transgene expression in a cancer-specific manner (28). Chen *et al* demonstrated that the survivin promoter can drive the expression of BikDD in lung cancer cells and inhibit cancer cell growth *in vitro* and *in vivo*(20). A high level of survivin expression has been observed in HCC (19). In the present study, four different plasmids were constructed with the survivin promoter. Following transfection with GFP/pSURV, a stronger fluorescence signal was observed in the HepG2 cells, but not in the L02 cells and TK gene expression observed in the HepG2 cells, but not in the L02 cells following transfection with TK/pSURV and TAT-TK/pSURV. These results suggest that when the survivin promoter-driven suicide gene is systemically administered, the systemic toxicity can be significantly reduced.

Besides the advantage of the survivin promoter, the HSV-TK/GCV suicide gene therapy system has another superiority: the ‘bystander effect’. The ‘bystander effect’ occurs when non-transfected or genetically unmodified cells are killed as the result of enzyme/prodrug activation during the death of genetically modified tumor cells transfected with a suicide gene. The ‘bystander effect’ greatly amplifies the efficacy of HSV-TK/GCV gene therapy for cancer in which only a fraction of the cells are targeted. Suicide gene therapy has become more effective with the enhancement of the ‘bystander effect’ (29). This phenomenon is thought to occur due to the transfer of toxic GCV metabolites from HSV-TK-modified cells to non-transfected tumor cells through gap junctions (30–32).

In this study, when fused with HIV-1 TAT, the ‘suicide gene’ product TK induced cell death with a significant ‘bystander effect’. Our results also indicated that induced apoptosis increased when the ‘suicide gene’ product TK was fused with HIV-1 TAT. It has been previously demonstrated that the cytotoxicity of GCV can be enhanced by fusing the HSV-TK with the cell penetrating peptide from the HIV-1 transactivator protein transduction domain (TAT PTD) (33). The mechanism involved however, remains unclear. Previous studies have focused on the intracellular transduction of TAT, as the GRKKR sequence of TAT can act as a nuclear localization signal (34). A substantial amount of research has been carried out using a 10–12 amino acid domain derived from the HIV TAT protein (35). This peptide enters cells by a non-receptor-dependent, non-energy-dependent mechanism that presumably involves passive permeation across the plasma membrane (34). This peptide also contains nuclear localization signals (NLS) that help to overcome the nuclear membrane bottleneck (36). Cao *et al* found that when the suicide gene TK was fused with TAT, the ‘suicide gene’ product TK bound to the plasma membrane and entered the hepatoma cells. Moreover, it was targeted to the nucleus and the internalized TK was stable (37).

In this study, we demonstrate the efficacy of the suicide gene therapy system driven by the survivin promoter in suppressing HCC cell growth and inducing apoptosis when the ‘suicide gene’ product TK was fused with HIV-1 TAT. Apoptosis, also known as programmed cell death, refers to certain physiological or pathological conditions in which the end of active life is regulated by the activation of a set of apoptotic factors. We detected the level of PCNA and cleaved caspase-3. In normal cells, apoptosis and proliferation co-exist and maintain a dynamic equilibrium. When the TK/GCV suicide gene targeting system was delivered into tumor cells, a significant inhibition of cell growth was observed, as well as enhanced apoptosis via the increased caspase-3 protein activation. Therefore, our findings strongly suggest that the survivin promoter-mediated tumor-targeting suicide gene therapy system may represent a novel therapy for HCC.
